# Spatial patterning of hypertension and its association with comorbidities and risk factors: A cross-sectional study in South India

**DOI:** 10.1371/journal.pone.0336252

**Published:** 2025-12-19

**Authors:** Thenmozhi Mani, Marimuthu Sappani, Melvin Joy, Chhavi Garg, Vishalakshi Jeyaseelan, Malavika Babu, Rajagopal Arunachalam, Jothilakshmi Durairaj, Ivan James Prithishkumar, Sebastian George, Edwin Sam Asirvatham, Thambu David Sudarsanam, Lakshmanan Jeyaseelan

**Affiliations:** 1 Population Health Research Institute, McMaster University, Hamilton, Ontario, Canada; 2 Department of Health Research Methods, Evidence and Impact, McMaster University, Hamilton, Ontario, Canada; 3 Translational and Clinical Research Institute, Faculty of Medical Sciences, Newcastle University, Newcastle Upon Tyne, United Kingdom; 4 Faculty of Science, Global Health Research, Vrije Universiteit, Amsterdam, the Netherlands; 5 Polio Eradication, World Health Organization, Geneva, Switzerland; 6 Centre for Trials Research, College of Biomedical and Life Sciences, Cardiff University, Cardiff, United Kingdom; 7 Department of Biostatistics, Christian Medical College, Vellore, Tamil Nadu, India; 8 Schizophrenia Research Foundation (SCARF), Chennai, India; 9 Mohammed Bin Rashid University of Medicine and Health Sciences, Dubai Healthcare City, Dubai, United Arab Emirates; 10 Department of Statistical Science, Kannur University, Kannur, Kerala, India; 11 Health Systems Research India Initiative (HSRII), Trivandrum, Kerala, India; 12 Department of Medicine, Christian Medical College and Hospital, Vellore, Tamil Nadu, India; Military University of Technology Faculty of Civil Engineering and Geodesy: Wojskowa Akademia Techniczna im Jaroslawa Dabrowskiego Wydzial Inzynierii Ladowej i Geodezji, POLAND

## Abstract

**Introduction:**

Non-communicable diseases (NCDs), particularly hypertension (HTN), pose a significant global health challenge, accounting for a substantial proportion of premature deaths worldwide. In India, HTN prevalence varies widely across states and districts and is influenced by demographic, socioeconomic, and lifestyle factors. This study aims to assess the spatial distribution of HTN and its correlates in South India.

**Materials and methods:**

This study utilized data from the 5th National Family Health Survey (NFHS-5), a nationally representative cross-sectional survey conducted across India between 2019 and 2021. For this analysis, data from five states and one union territory in South India were used. Bayesian spatial modelling was employed to analyse HTN prevalence at the district level, incorporating demographic, socioeconomic, and lifestyle covariates.

**Results:**

The study included 304,420 adults, both male and female, aged >18 years. The overall prevalence of pre-HTN and HTN was 28.9% and 31.8%, respectively. HTN prevalence varied across states, with Kerala exhibiting the highest prevalence. Spatial clustering analysis identified districts with significantly higher HTN prevalence, often clustering with neighbouring districts showing similar patterns. Spatial autocorrelation analyses revealed a significant association between HTN and diabetes. Other comorbidities and risk factors were not significantly associated with HTN.

**Conclusion:**

The findings underscore the spatial heterogeneity of HTN prevalence within South Indian states and districts. The study highlights the need for targeted interventions tailored to local contexts to effectively mitigate the burden of HTN and associated comorbidities.

## Introduction

Non-communicable diseases (NCDs) remain a significant global public health challenge, with an increasing burden in low and middle-income countries. Nearly 41 million people die each year due to NCDs, accounting for 74% of all deaths globally [1]. Hypertension (HTN) is a significant and modifiable risk factor for cardiovascular, renal, and stroke diseases and a major cause of premature death [2,3]. HTN alone is associated with 7.5 million deaths and 12.8% of all deaths globally [4].

According to the National Family Health Survey (NFHS) in 2016, the age-adjusted HTN prevalence in India was 11.3% (95%CI: 11.1, 11.4) among individuals aged 15–49 years (men 13.8%, women 10.9%). The survey also found that 35.6% of men aged 25–54 years had HTN (urban 38.4%, rural 33.8%) [5]. Another study in India indicated a hypertension prevalence of 30.7% among the adult population in 2015, with only 12.9% on antihypertensive medication [6]. Geldsetzer et al (2018) reported a hypertension prevalence of 25.3% among the adult population in India, with a significantly higher rate in urban areas (32.5%) [7]. Several studies in India have indicated significant variations in the prevalence of HTN between and within states [8].

Hypertension often coexists with obesity, diabetes, anaemia, hyperlipidaemia, or metabolic syndrome [9]. Prenissl et al (2022) observed that the three most prevalent comorbidities with hypertension were obesity (2.9%), anaemia (2.2%), and obesity with anaemia (1.2%) [10]. Common risk factors related to HTN are age, education, income or wealth index, gender, marital status, type of family, family history of hypertension, tobacco chewing, stress, and body mass index (BMI) [11]. Lifestyle behaviours such as smoking, alcohol consumption, and dietary habits are also associated with the risk of developing HTN and diabetes [9]. Ragavan et al (2023) reported a two-fold higher risk for hypertension in people who were overweight according to both BMI and measures of central adiposity [12].

The Government of India (GoI) has adopted the “25 by 25” goal, which aims to reduce premature mortality due to NCDs by 25% by 2025. One of the key targets is to reduce the prevalence of high HTN by 25% [13]. The Indian Hypertension Control Initiative (IHCI), a 5-year initiative of the GoI, aims to implement evidence-based strategies to strengthen the building blocks of hypertension management and control [14]. Understanding the geographical distribution and its variations in the burden of HTN, associated morbidities, and contributory risk factors is critical for healthcare planning and equitable service delivery. Geographic Information Systems (GIS) based disease mapping is a valuable tool in public health planning and developing disease control strategies by providing a comprehensive understanding of the relationship between disease and its geographic distribution [15].

GIS allows visualization of disease occurrence locations, observation of spread patterns, and identification of risk factors associated with the disease spread after adjusting for spatial clustering. Spatial analysis techniques are also useful in defining exposure-outcome relationships for understanding health aspects exhibiting spatial phenomena. Although several studies have addressed the prevalence of HTN in India and its associated risk factors, few have focused on spatial clustering. However, none of them looked at the spatial association of HTN with other risk factors and spatial regression models [8,16].

The south Indian states are culturally distinct and relatively well-developed, with better health systems and health outcomes compared to other regions. However, emerging evidence points to an increasing burden of non-communicable diseases (NCDs) such as diabetes, hypertension, and obesity, warranting further investigation. Given the importance, this study examines the spatial distribution of HTN and the spatial correlation of common risk factors within South Indian states.

## Materials and methods

### Study design

We analysed data from the 5^th^ National Family Health Survey (NFHS) of India, conducted on representative samples of households across the country between 2019 and 2021. The data was curated from https://dhsprogram.com/data/dataset_admin. This cross-sectional survey collected detailed information on population, health, and nutrition. We included adults aged ≥18 years from five states, namely Andhra Pradesh (AP), Karnataka, Kerala, Telangana, and Tamil Nadu (TN), and Puducherry, a union territory (UT), in the analysis.

### Independent and dependent variables

The analysis included a range of socioeconomic, demographic, and behavioural covariates as independent variables, including age, gender, place of residence, diabetes mellitus (DM), smoking status, alcohol, poverty, obesity, waist-hip (WH) ratio, and marital status. The dependent variable was systolic and diastolic BP.

Among independent variables, obesity was categorized as a binary variable (yes or no) based on BMI ≥ 30.0 kg/m^2^ or BMI < 30.0 kg/m^2^ [17]. The wealth index was dichotomized by combining the “poorest” and “poor” categories into a single “poor” group; all other categories were combined as others. Diabetes status was determined using random blood glucose measurements, obtained through finger-stick blood specimens from individuals aged 15 and above using the Accu-Chek Performa glucometer. DM was classified as ‘yes’ or ‘no’ based on the blood glucose level. Glucose levels <30 mg/dL were excluded from the analysis as unreliable. The individual was considered diabetic if either fasting glucose was ≥ 126 mg/dL or random blood glucose was ≥ 200 mg/dL, or had been indicated as having a high glucose level two or more times by a doctor or other health professionals, as per the recommendations of the International Diabetes Federation and WHO [18].

For the dependent variable – systolic and diastolic BP, three readings were recorded during a single occasion. The average of the 2^nd^ and 3^rd^ measurements was considered the final BP value. If either the second or third reading was absent, the first reading was used to calculate the average. If fewer than two readings were available, the individual’s BP value was considered missing. HTN was defined as having an average blood pressure of ≥140 mmHg in the systolic or ≥90 mmHg in the diastolic range [19]. An average diastolic blood pressure of 80 to 89.99 mmHg and an average systolic blood pressure of 130 to 139.99 mmHg were considered pre-HTN [20]. A person was considered to have hypertension (HTN) if their systolic or diastolic blood pressure was higher than normal or if they had been told they had high blood pressure two or more times by a physician or other healthcare provider. Systolic values below 70 mmHg and diastolic values below 20 mmHg were considered missing BP values.

### Inclusion and exclusion criteria

The study included 3,04,420 participants aged 18 and above, both male and female, and excluded the participants’ children and adolescents.

### Statistical analysis

We conducted a spatial analysis to assess the clustering of hypertension across districts in South Indian states. The weighted prevalence was calculated using the survey weights given by the demographic and health surveys (DHS). Specifically, the inverse of the household selection probability was used as a weight. First, we evaluated global spatial dependence using Global Moran’s I index on weighted hypertension prevalence rates. Moran’s I range from −1 to +1, with values near zero indicating random spatial distribution, positive values indicating spatial clustering, and negative values suggesting spatial dispersion, where neighbouring values are dissimilar. Once global spatial autocorrelation was established, we applied the Local Indicators of Spatial Association (LISA) to identify and quantify spatial clusters at the district level [21]. This method enabled us to compare hypertension rates in each district against those of neighbouring districts and visually represent spatial dependencies. Four distinct spatial patterns emerged: high/high (districts with high rates surrounded by similarly high-rate districts), low/low (districts with low rates surrounded by low-rate districts), high/low, and low/high (districts where high rates are bordered by low-rate districts and vice versa). The high/high and low/low categories identify areas of concordance, while high/low and low/high signify transition areas.

To explore associations between HTN and related risk factors, we used Bivariate LISA analysis. This approach tested the hypothesis that districts with high HTN prevalence are spatially associated to districts with high prevalence of HTN and diabetes (HTN-DM) and other risk factors, such as obesity, alcohol consumption, and smoking. For instance, a high-high bivariate cluster would represent a region with both high HTN prevalence and a high rate of HTN-DM, or similarly high levels of obesity, alcohol use, and smoking.

The Bayesian Beta regression model was developed by considering the proportion of HTN as the dependent variable, which incorporated the spatial correlation. The Integrated Nested Laplace Approximation (INLA) was used to estimate the regression coefficients with the following priors:

1. The prior for the intercept followed a Gaussian distribution with a mean of 0 and an infinite variance (precision of 0).
2. The priors for the slope coefficients were also Gaussian, with a mean of 0 and a precision of 0.001.
3. The prior for the precision (inverse variance) of the random intercept was specified as a Gamma distribution with shape and rate parameters of 1 and 0.00005, respectively.

To model the spatial component, the precision matrix was constructed using the stochastic partial differential equation (SPDE) approach. A spatial mesh was created based on the longitude and latitude coordinates, which was then used to construct the projection matrix. The spatial field was modelled using the Matérn SPDE framework. Finally, a stack object was created by combining the projection matrix, spatial field, and the data, setting up the model for estimation. We first developed a univariate beta regression model, followed by multivariable models incorporating all clinically important risk factors. The posterior median along with 95% credible interval (CrI) was reported. Model performance and goodness of fit statistic were evaluated using marginal likelihood, the Deviance Information Criterion (DIC), and the Watanabe-Akaike Information Criterion (WAIC). The Bayesian Beta regression analysis was done using R-INLA (http://www.r-inla.org/). Choropleth and LISA maps were done using QGIS 3.38.1 and Geoda 1.22.0.4 software [22,23].

### Ethical considerations

The study was based on secondary data analysis with the dataset available in the public domain for open use. The analysis was approved by the Institutional Review Board of Christian Medical College (CMC), Vellore, India.

## Results

The study included 304,420 participants aged 18 and above across five South Indian states and a Union Territory with the following distribution: Andhra Pradesh (10.0%), Kerala (11.5%), Karnataka (27.6%), Puducherry (3.3%), Telangana (24.2%), and Tamil Nadu (23.4%). The majority were from rural areas (65%), and 53% were female.

**Fig 1:**
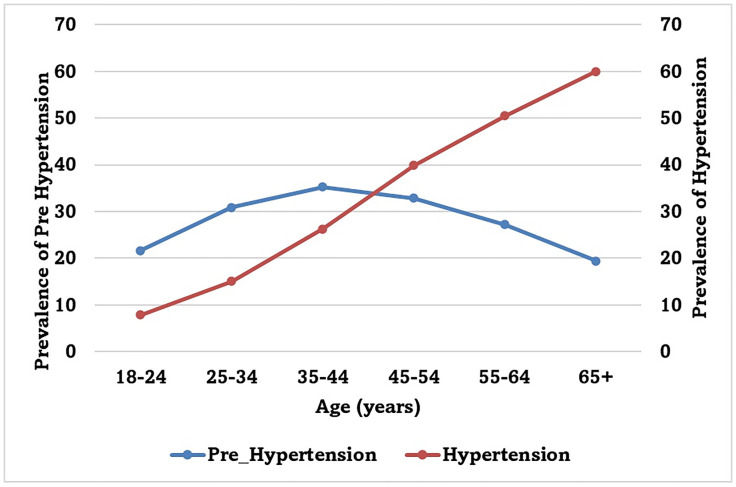
Prevalence of Pre-Hypertension and Hypertension by Age (years).

The estimated weighted prevalence of hypertension (HTN) in South India was 31.8% (95% CI: 31.7, 32.0), ranging from 28.8% in Puducherry to 35.3% in Kerala. HTN was higher in males (32.8%) than in females (31.0%), and more prevalent in urban (33.9%) than in rural areas (30.5%) (Table 1). Prevalence of HTN increased with increasing age (Fig 1).

**Table 1:** Weighted prevalence of Pre-Hypertension and Hypertension by state, age, gender, and Residence.

	Overall Total*	Pre HTN		HTN	
		Yes	Proportion (95% CI)	Yes	Proportion (95% CI)
**Overall** **(South India)**	417227	104927	28.9 (28.7 - 29.0)	115682	31.8 (31.7 - 32.0)
Andhra Pradesh	71531	19918	27.8 (27.5 - 28.2)	21572	30.2 (29.8 - 30.5)
Karnataka	84883	25393	29.9 (29.6 - 30.2)	26456	31.2 (30.9 - 31.5)
Kerala	49868	13586	27.2 (26.9 - 27.6)	17580	35.3 (34.8 - 35.7)
Tamil Nadu	113419	33526	29.6 (29.3 - 29.8)	36108	31.8 (31.6 - 32.1)
Puducherry	1777	520	29.2 (27.2 - 31.4)	512	28.8 (26.7 - 31.0)
Telangana	41901	11984	28.6 (28.2 - 29.0)	13454	32.1 (31.7 - 32.6)
**Gender**					
Female	197896	49436	25.0 (24.8 - 25.2)	61439	31.0 (30.8 - 31.3)
Male	165485	55491	33.5 (33.3 - 33.8)	54243	32.8 (32.6 - 33.0)
**Residence**					
Rural	218285	63078	28.9 (28.7 - 29.1)	66544	30.5 (30.3 - 30.7)
Urban	49138	41849	28.8 (28.6 - 29.1)	49138	33.9 (33.6 - 34.1)
**Age (years)**					
18 - 24	50202	10938	21.8 (21.4 - 22.2)	3976	7.9 (7.7 - 8.2)
25 - 34	75101	23235	30.9 (30.6 - 31.3)	11370	15.1 (14.9 - 15.4)
35 - 44	72153	25492	35.3 (35.0 - 35.7)	18998	26.3 (26.0 - 26.7)
45 - 54	64560	21247	32.9 (32.5 - 33.3)	25760	39.9 (39.5 - 40.3)
55 - 64	55007	14985	27.2 (26.9 - 27.6)	27753	50.5 (50.0 - 50.9)
≥ 65	46358	9030	19.5 (19.1 - 19.8)	27825	60.0 (59.6 - 60.5)

*Weighted counts.

**Fig 2:**
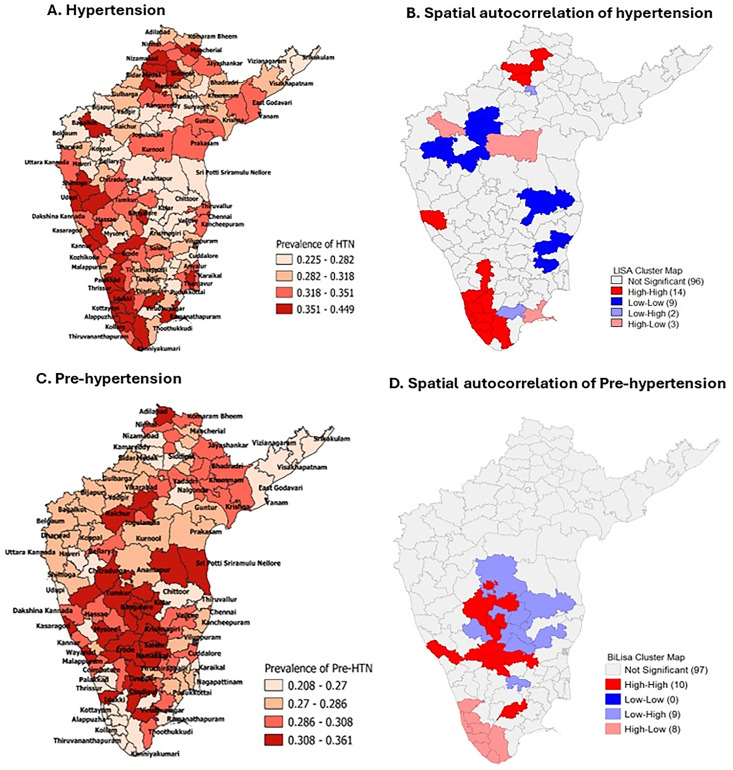
District-wise weighted prevalence and spatial autocorrelation (A) Hypertension. (B) Spatial autocorrelation of hypertension. (C) Pre-hypertension. (D) Spatial autocorrelation of Pre-hypertension.

The estimated weighted prevalence of pre-hypertension (pre-HTN) was 28.9% (95% CI: 28.7, 29.0), which varies from 27.2% in Kerala to 29.9% in Karnataka. Pre-HTN was more common in males (33.5% vs 25.0%) and was almost similar in rural and urban areas (28.9% vs 28.8%). Younger adults (18–25 years) had a lower pre-HTN prevalence (21.8%) compared to those aged 35–44 years (35.3%) (Table 1). Pre-HTN prevalence increased with age till 44 years and declined after that. A significant proportion transitioned to hypertension with increasing age (Fig 1). The four quartiles of estimated HTN prevalence in South India are shown in Fig 2.

### Spatial clustering of HTN prevalence

The spatial clustering of HTN in various states is depicted in Fig 2. Karnataka had 23% (7/30) of the districts with a high prevalence of HTN; of these, three districts (Shimoga, Udupi, and Chikmagalur) are adjacent to each other. Telangana had 22.6% (7/23) of districts with a high prevalence of HTN, with four neighbouring districts (Nizamabad, Rajanna Sircilla, and Sangarreddy). Tamil Nadu had 18.7% (6/32) of districts with a high prevalence of HTN, and two districts were adjacent (Kanyakumari and Tirunelveli). Kerala had the highest proportion of districts with increased HTN levels (64%; 9/14). Puducherry Union Territory had one district with high a prevalence of HTN, whereas Andhra Pradesh did not have any HTN high-prevalence districts or clustering of such districts (Fig 2).

### Spatial clustering between pre-HTN and HTN

Of the 124 districts in South India, 10 (8.1%) districts exhibited a clustering of high-prevalence HTN districts, surrounded by high-prevalence pre-HTN districts. The distribution in each state is as follows: Tamil Nadu had demonstrated three districts (Madurai, Erode, and Salem); two from Kerala (Kannur and Wayanad), and five from Karnataka (Chamarajanagar, Ramanagar, Bangalore, Tumkur, and Chikkaballapura). Nevertheless, the global statistic for assessing the clustering of HTN with pre-HTN was found to be nearly zero (−0.095; p = 0.019), indicating almost no association (no spatial clustering of districts) between pre-HTN and HTN (Fig 2, Table 2). Also, the association between Pre-Hypertension and Hypertension (tertile) for five states is presented in Table 3.

**Table 2:** Spatial correlation (Moran’s I) and clustering with Hypertension.

Variables	Global Moran’s I	Z value	P value	Hypertension (N = 124)			
				High- High n (%)	Low-Low n (%)	High – Low n (%)	Low – High n (%)
**HTN (Univariate)**	0.287	4.96	0.001	Total: 14 (0.11) TN(2):Tirunelveli, Kanyakumari Kerala(7):Ernakulam, Idukki,Kottayam, Alappuzha, Pathanamthitta, Kollam, Thiruvananthapuram Karnataka(1): Dhaksina Kannada Telangana(4): Nizamabad,Rajanna Sircilla, Siddipet,Jagtial	Total: 9 (0.07) TN (3): Villupuram, Peramballur, Ariyalur Karnataka(5): Dharwad,Gadag, Bellary,Raichur, Yadgir AP (1): Chittoor	Total: 3 (0.02) TN(1): Ramanathapuram Telangana(1): Bagalkot AP (1): Kurnool	Total: 2 (0.02) TN(1): Virudhunagar Telangana(1): Medchal
**Pre-Hypertension**	−0.095	−2.162	0.019	Total: 10 (0.08) TN (3): Madurai, Erode, Salem Kerala(2):Kannur, Wayanad Karnataka(5): Chamarajanagar, Ramanagar, Bangalore, Tumkur, Chikkaballapura	Total: 0 (0%)	Total: 8 (0.06) TN(2):Tirunelveli, Kanyakumari Kerala(6): Thiruvananthapuram, Kollam, Pathanamthitta, Alappuzha, Kottayam, Ernakulam	Total: 9 (0.07) TN(4):Karur, Dharmapuri, Tiruvannamalai, Krishnagiri Karnataka(3): Kolar,Mandya, Bangalore rural AP(2):Chittor, Anantapur
**Obese**	0.069	1.658	0.043	Total: 10 (0.08) TN(7):Tirunelveli, Nagapattinam, Tiruvarur, Kanchipuram, Chennai, Thiruvallur, Erode Kerala(3): Thiruvananthapuram, Idukki, Palakkad.	Total: 14 (0.11) Karnataka(6): Gulbarga,Yadgir, Raichur,Koppal, Bijapur, Dharwad, Telangana(8): Jogulamba, Wanaparthy, Mahabubnagar, Adilabad, Karimnagar, Warangal, Warangal rural, Jangaon	Total: 12 (0.1) Kerala (2): Udupi, Kannur Karnataka(3): Bagalkot, Kurnool, Kodagu Telangana(7): Mancherial, Jayashankar Bhupalpally, Jatigal,Rajanna Sricilla, Siddipet, Kamareddy, Nizamabad	Total: 6 (0.05) TN(5): Virudhunagar, Dindigul, Karur, Vellore, Villupuram, Puducherry(1): Karaikal
**DM**	0.249	5.43	0.001	Total: 10 (0.08) TN(3):Tirunelveli, Kanyakumari and Theni Kerala(7):Ernakulam, Idukki,Kottayam, Alappuzha, Pathanamthita,Kollam and Thiruvananthapuram	Total: 18 (0.15) Karnataka(12): Belgaum, Dharwad,Haveri, Davanagere, Bellary,Koppal, Gadag,Raichur, Yadir, Mahabubnagar, Bijapur, Gulbarga Telangana (2): Jogulamba Gadwal, Wanaparthy AP (4): Anantapur, Adilabad, Warangal urban, Warangal rural	Total: 16 (0.13) Kerala (1): Udupi Karnataka(7): Bagalkot Chikmagalur, Shimoga, Uttara Kannada, Chitradurga, Tumkur, Ramanagar, Telangana(7): Vikarabad, Sangareddy, Kamareddy, Nirmal,Jatigal, Mancherial, Jayashankar Bhupalpally AP (1): Kurnool	Total: 5 (0.04) TN(5): Thoothukudi, Virudhunagar, Sivaganga, Thanjavur, Tiruchirappalli
**Alcohol**	−0.01	−0.23	0.422	Total: 13 (0.1) Telangana(12): Mancherial, Jayashankar, Peddapalli,Jagital, Nizamabad, Kamareddy,Rajanna Sricilla,Siddipet, Medchal,Sangareddy, Vikarabad, Ranga reddy AP (1): Guntur	Total: 14 (0.11) TN (1): Krishnagiri Kerala(1): Kasargod Karnataka(10): Bijapur,Belgaum, Dharwad,Gadag, Haveri, Davanagere, Bellary,Koppal, Kolar,Bangalore rural AP (2): Anantapur, Chittoor	Total: 17 (0.14) TN(3): Kanyakumari, Tirunelveli,The Nilgiris Kerala(4): Kozhikode, Thiruvananthapuram,Udupi, Wayanad Karnataka(9): Kodagu, Ramanagara, Tumkur, Chikkaballapura, Chitradurga, Chikmagalur, Shimoga,Uttara Kannada, Bagalkot Puducherry(1): Mahe	Total: 13 (0.1) Telangana (13): Bhadradri, Mahabubad Suryapet, Nagalgonda, Nagar Kurnool, Wanaparthy, Mahabubnagar, Yadadri, Medchal, Jangaon, Warangal rural, Karimnagar, Warangal urban
**Smoke**	−0.219	−5.14	0.001	Total: 7 (0.06) Karnataka(6):Uttara Kannada,Shimoga, Chikmagalur, Chitradurga,Tumkur, Bagalkot, Telangana(1): Mancherial	Total: 2 (0.02) TN(2): Thoothukkudi, Virudhunagar	Total: 14 (0.11) TN(4): Tirunelveli, Kanyakumari, Kanchipuram, Tiruvallur Kerala(9): Thiruvananthapuram,Kollam, Alappuzha, Pathanamthitta, Kottayam, Idukki, Thirussur, Kozhikode, Wayanad Puducherry(1): Mahe	Total: 13 (0.1) Karnataka(11): Gulbarga, Yadgir, Raichur, Bellary, Davanagere, Haveri, Belgaum, Bijapur, Dharwad, Gadag, Koppal Telangana(2): Adilabad, Mahabubnagar
**Poverty**	−0.304	−6.93	0.001	Total: 5 (0.04) TN (1): Tanjavur Karnataka (1): Bagalkot Telangana(3): Mancherial, Kamareddy,and Sangareddy	Total: 3 (0.02) Kerala(2): Kasaragod, Malappuram Karnataka(1): Bangalore rural	Total: 18 (0.15) TN(4): Tirunelveli, Coimbatore, Tiruppur,The Nilgiris Kerala(12): Thiruvananthapuram,Kollam, Pathanamthitta, Alappuzha,Ernakulam, Kottayam, Idukki, Thirissur, Kozhikode, Wayanad, Kodagu, Kannur Puducherry(1): Mahe Telangana(1): Hyderabad	Total: 13 (0.1) Karnataka(11): Bidar, Gulbarga, Yadgir, Mahabubnagar, Raichur, Bellary, Koppal, Gadag, Dharwad, Belgaum, Bijapur Telangana(2): Adilabad, Jogulabma
**Mean Age**	0.288	6.53	0.001	Total: 15 (0.12) TN(4):Tirunelveli, Coimbatore, Erode and Thanjavur Kerala(11):Kannur, Kozhikode,Thrissur, Ernakulam,Idukki, Kottayam,Alappuzha, Pathanamthitta, Kollam, Thiruvananthapuram Puducherry (1): Mahe	Total: 16 (0.13) Karnataka(10): Bidar,Gulbarga, Bijapur,Yadgir, Raichur,Koppal, Bellary,Gadag, Belgaum, Haveri Telangana(6): Mahabubnagar, Nagarkurnool, Jogulamba, Wanaparthy, Yadadri, Medchal	Total: 10 (0.08) Karnataka(2): Kurnool, Bagalkot Telangana(7): Kamareddy, Medchal, Siddipet, Sangareddy, Rangareddy, Vikarabad, Hyderabad AP (1): East Godawari	Total: 4 (0.03) TN(3): Cuddalore, Ariyalur,and Tiruchirappalli Kerala(1): Malappuram
**Mean WH Ratio**	0.269	6.23	0.001	Total: 18 (0.15) TN(4):Tirunelveli, Theni,Madurai, Coimbatore Kerala(12): Thiruvananthapuram, Kollam,Idukki, Alappuzha,Pathanamthitta, Kottayam,Ernakulam, Thrissur,Palakkad, Kozhikode,Wayanad, Kannur Karnataka (1): Kodagu Puducherry (1): Mahe	Total: 15 (0.12) Karnataka(9): Haveri,Dharwad, Belgaum,Gadag, Koppal,Bellary, Raichur,Yadgir, Bijapur Telangana(4): Mahabubnagar, Wanapathy, Adilabad, Karimnagar AP (1): Anantapur	Total: 11 (0.09) Karnataka(1): Bagalkot Telangana(9): Mancherial, Jagtial, Peddapalli, Nizamabad, Rajanna Sircilla, Kamareddy, Sangareddy, Siddipet, Medchal AP (1): Kurnool	Total: 6 (0.05) TN(5): Virudhunagar, Dindigul, Tiruppur, Karur, Tiruchirappalli Kerala(1): Malappuram

**Table 3:** Association between Pre-Hypertension and Hypertension for five states.

		Proportion of HTN (Tertile)		
		Mild (n = 41)	Moderate (n = 41)	High (n = 42)
**Proportion of Pre-HTN (Tertile)**	**Mild**	11 (26.8%)	7 (17.1%)	23 (56.1%)
	**Moderate**	16 (39.0%)	18 (43.9%)	7 (7.1%)
	**High**	14 (33.3%)	16 (38.1%)	12 (28.6%)

### Spatial clustering of comorbidities (DM and Obesity) and HTN

**Fig 3:**
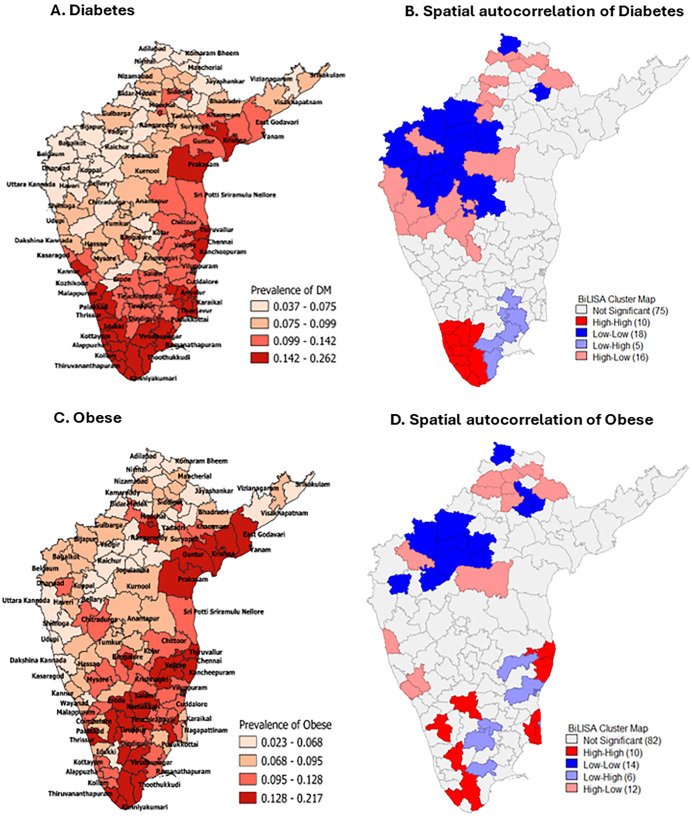
District-wise weighted prevalence and spatial autocorrelation. (A) Diabetes. (B) Spatial autocorrelation of Diabetes. (C) Obese. (D) Spatial autocorrelation of Obese.

Fig 3A and 3B, and Table 2 illustrate the geographical distribution of the prevalence of Diabetes Mellitus (DM) and the spatial autocorrelation of HTN with DM, respectively. The data indicated that 28 districts exhibited a high prevalence of HTN with a high prevalence of DM (High-High). Specifically, 7 and 3 districts in Kerala and Tamil Nadu, respectively, showed High clustering of HTN and DM districts. The spatial correlation between HTN and DM was 0.249 (p = 0.001), suggesting a significant spatial clustering of districts concerning HTN and DM. The spatial distribution of obesity and the spatial clustering between HTN and obesity are depicted in Fig 3C and 3D, respectively, and Table 2. Spatial clustering between the high prevalence of HTN and obesity (High-High) was identified in 10 districts. The spatial autocorrelation value between HTN and obesity is 0.069 (p = 0.043), indicating a very low spatial correlation.

**Fig 4:**
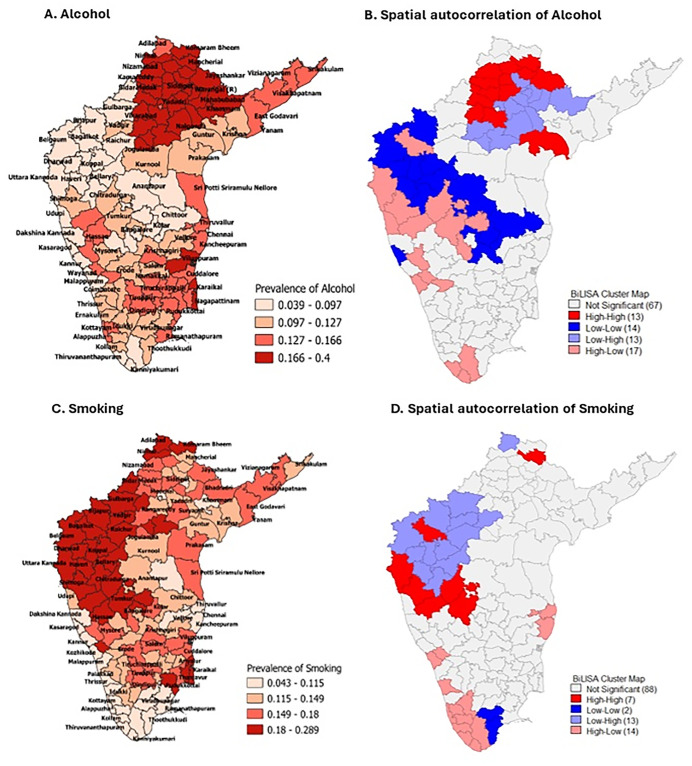
District-wise weighted prevalence and spatial autocorrelation (A) Alcohol. (B) Spatial autocorrelation of Alcohol. (C) Smoking. (D) Spatial autocorrelation of Smoking.

Similarly, the spatial clustering of HTN with risk factors – the proportion of alcohol drinkers and smokers- was assessed and is presented in Fig 4. The results indicate no spatial association between alcohol consumption and HTN. Conversely, there was a significant spatial clustering with age, smoking, WH ratio, and poor population.

### Multivariable regression adjusting for spatial correlation

Posterior median with 95% CrI of both unadjusted and adjusted regression were presented in Table 4. In the unadjusted analysis, the mean age and proportion of DM were positively associated with the proportion of hypertension. In contrast, the proportion of poor was negatively associated (posterior median of −0.489 (95% CrI: −0.805, −0.156)) with hypertension. In adjusted analysis, proportion of DM was significantly associated with hypertension, posterior median of 2.567 (95% CrI: 1.277, 3.884). A district with a high prevalence of DM may also have a high prevalence of hypertension.

**Table 4:** Bayesian Beta regression analysis adjusted for spatial correlation and risk factors of hypertension.

Variables	Univariate Model	Multivariable Model
	Posterior Median (95% CrI)	Posterior Median (95% CrI)
Mean Age	0.033 (0.007, 0.057)	0.011 (−0.022, 0.043)
Proportion of Smokers	−0.749 (−1.644, 0.183)	1.119 (−0.240, 2.460)
Proportion of (drinkers) Alcohol	0.071 (−0.623, 0.757)	0.457 (−0.231, 1.146)
Proportion of Obese	0.933 (−0.073, 1.946)	−0.212 (−1.594, 1.188)
Proportion of Urban residence	0.147 (−0.019, 0.313)	0.070 (−0.203, 0.345)
Proportion of DM	2.522 (1.499, 3.618)	2.567 (1.277, 3.884)
Proportion of Male	−0.285 (−3.143, 2.562)	0.128 (−3.042, 3.332)
Mean WH Ratio	1.737 (−1.151, 4.387)	0.049 (−2.702, 2.805)
Proportion of Marital Status	−0.99 (−2.59, 0.614)	−0.805 (−2.778, 1.178)
Proportion of Poor	−0.489 (−0.805, −0.156)	−0.324 (−0.832, 0.216)

CrI: Credible Interval; DIC=-465.94; WAIC=-472.91; Marginal likelihood=175.19.

## Discussion

Our study is the first to examine the spatial clustering between HTN and its comorbidities, specifically diabetes, obesity, and risk factors such as smoking and alcohol consumption, in relation to its neighbouring districts within South India. While similar spatial analyses have been conducted in other countries with comparable objectives, this study offers newer insights within the Indian context, particularly at the subnational level. The comorbidities and risk factors included in the study were selected due to their high prevalence and programmatic importance in the southern states. From 2019 to 2021, the overall prevalence of pre-HTN and HTN in South India was 28.9% and 31.8% respectively, higher than the national average. Ramakrishnan et al.(2015) reported a HTN prevalence of 30.7% [6]. A study by Geldsetzer et al (2008) reported an HTN prevalence of 32.6% among females and 32.4% among males, with notably higher rates observed in the southern Indian states compared to the national average [7] Our study indicated mild variations in the HTN prevalence between states but significant variations between districts, likely due to varying diagnostic, treatment, and control practices at the district level [8].

The study found a DM prevalence of 11.9% similar to 11.4% reported in another national cross-sectional study [24]. However, significant state-level variations were noted, with 17.7% in Kerala and 14.7% in Tamil Nadu. Among those with HTN, DM prevalence was 33.7% in Kerala and 31.7% in Tamil Nadu, indicating that approximately one-third of the adult population in these states has both conditions. Previous observational studies have confirmed the association between type 2 diabetes mellitus (T2D) and increased risk of HTN [7]. This was also evident from the clustering observed between DM and HTN.

Kerala exhibited the highest spatial correlation between high levels of HTN and DM (0.36) compared to 0.06 and 0.08 in Tamil Nadu and Andhra Pradesh, respectively. This could be due to Kerala’s older population and higher socio-economic status [25]. Our results suggest that districts with high HTN prevalence are more likely to have neighbouring districts with high DM levels, especially in Tamil Nadu and Kerala, which call for targeted interventions to manage the dual burden of HTN and DM. At the same time, other potential factors such as changing demography, environment, dietary patterns, and industries need to be investigated further to make a logical conclusion of the association.

In Tamil Nadu, 13 districts had a high prevalence of obesity, followed by 4 districts in Karnataka. There was minimal or no spatial correlation between high HTN levels and neighbouring districts with high obesity levels (Moran’s I = −0.084). The multivariable analyses did not indicate an association between obesity and HTN; however, several studies reported an association between Obesity and HTN [5,26,27]. This could be due to the primary focus of this paper, which is identifying geographic patterns and impacts, compared to other research that mainly focused on risk factor analysis.

No spatial association was found between alcohol consumption and HTN. However, Telangana state had the highest number of districts with high alcohol consumption, followed by Tamil Nadu’s coastal districts, warranting further investigation to explore the potential reporting biases regarding alcohol intake.

Karnataka had higher levels of smoking as compared to other states. Intensive smoking prevention and cessation interventions are required in districts with high smoking and high HTN prevalence. The overall spatial autocorrelation was −0.219, indicating a negative association between high HTN prevalence and smoking. Literature on the impact of tobacco use on the risk of hypertension is inconclusive; some studies suggest that tobacco use increases the risk of developing hypertension [28,29], while others indicate that current smoking is linked to similar or lower blood pressure levels [30,31]. A study by Devi et al (2013) reported that the life-course impact of smoking on hypertension was not statistically significant in individuals younger than 35, though it was significantly associated with HTN in the older age groups [32]. Another potential reason for the negative correlations between HTN and smoking could be the social stigma associated with smoking, leading to underreporting [33].

## Limitations

As the study included only South Indian states, which are relatively well-developed states with better health systems and health outcomes, the findings may not be representative of the entire population in India. In addition, the BP measurements were taken on one occasion only, though BP measurements are required to be taken on at least two different occasions to clinically diagnose hypertension, which may lead to overestimation.

Though the present study highlights the district-level spatial patterns of HTN and associated factors, it could not explore the causal factors of the spatial pattern and its variations. Some associations need further studies to explain it better and draw conclusions.

## Conclusion

This study provides evidence on the spatial epidemiology of HTN across districts in Southern India. The overall prevalence of HTN (29.9%) and pre-HTN (31.8%) exceeds the national average, underscoring the urgency for regional-specific public health response. Notably, Kerala and Karnataka states had the highest number of districts with higher prevalence of HTN, while Tamil Nadu state had a higher number of districts with high prevalence of Obesity, further adding to the overall cardiac health concerns in the region. The spatial clustering of HTN and DM, particularly in the districts of Kerala and Tamil Nadu, reveals an interlinked health challenge that demands integrated intervention strategies. Bayesian multivariable regression analysis confirmed a significant positive association between DM and HTN, which reinforces the need for data-driven health policies that prioritize high-burden districts and address overlapping risks. Since health is a state subject in India, our district-level spatial analyses offer relevant insights to individual states to develop geographically targeted and context-specific interventions, towards achieving the Government of India’s target of reducing the prevalence of high HTN by 25% by 2025.
